# Response: Commentary: Directions for Optimization of Photosynthetic Carbon Fixation: RuBisCO’s Efficiency May Not Be So Constrained After All

**DOI:** 10.3389/fpls.2019.01426

**Published:** 2019-11-22

**Authors:** Peter L. Cummins, Babu Kannappan, Jill E. Gready

**Affiliations:** John Curtin School of Medical Research, The Australian National University, Canberra, ACT, Australia

**Keywords:** RuBisCO, carbon fixation, photosynthesis, enzyme kinetics and specificity, protein evolution, evolutionary constraints, enzyme-complex stability, gas-substrate binding

## Introduction

Understanding the molecular mechanisms that make enzymes work remains one of the grand challenges in contemporary biophysics. If this understanding can be translated into the successful re-engineering of enzymes with greater efficiency, the practical benefits could be enormous. One such enzyme that has been targeted for re-engineering, ribulose 1,5-bisphosphate carboxylase/oxygenase (Rubisco, EC 4.1.1.39), is of intensive interest in agriculture and related fields as it fixes CO_2_ in higher plants and the vast majority of other photosynthetic organisms. In a recent article, Cummins et al. (2018a), we presented a statistical analysis of a wide range of published kinetic data on Rubisco. The results of that study suggested evidence of significant rates of decarboxylation (reaction with CO_2_) and deoxygenation (from the side reaction of Rubisco with O_2_) among wild-type Rubiscos. These results have challenged the accepted view that dissociation of the gas molecules (decarboxylation and deoxygenation) from the enzyme complex is negligible in all wild-type Rubiscos. In a commentary on Cummins et al. (2018a), Tcherkez et al. (2018) have contested our conclusion of significant decarboxylation and deoxygenation rates in Rubisco and suggested it is based on a misinterpretation of “implicit relationships between Rubisco rate constants” and “overlooks experimental evidence for feeble rates of deoxygenation and decarboxylation.” In this response to their commentary on Cummins et al. (2018a), we address these criticisms.

## Interpretation of Rate Constants

Firstly, it is necessary to clarify the significance of *γ*, a parameter which arises from explicitly including the rate of product release in the kinetic scheme (**Figure 1** in Cummins et al., 2018a). The equation for carboxylation is given by (see Supplementary Material in Cummins et al., 2018a but expressed in the form *x*/(*y* + *x*)),

**Figure 1:**
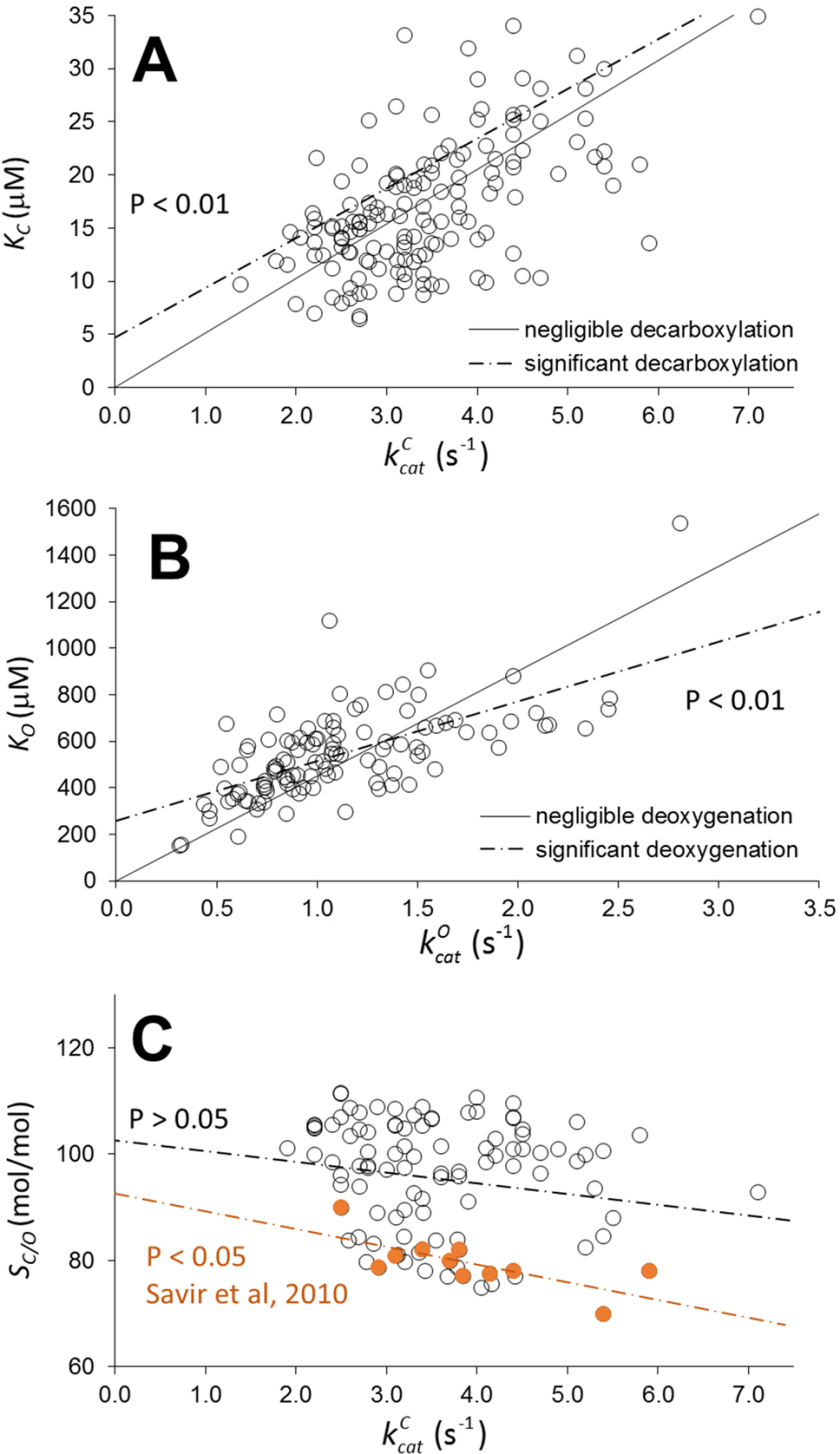
Scatter plots of *K_C_* as a function of $k_{cat}^{C}$ **(A)**, *K*_O_ as a function of $k_{cat}^{C}$ **(B)**, and relative specificity, *S_C_*_/0_, as a function of $k_{cat}^{O}$ **(C)** for wild type in higher-plant Rubiscos from various data compilations (Ishikawa et al., 2009; Galmés et al., 2014; Orr et al., 2016; Prins et al., 2016; Cummins et al., 2018a). Assuming decarboxylation and deoxygenation are negligible in all Rubiscos, the solid lines (with zero intercept) were obtained by optimizing only the coefficients of $k_{cat}^{C}$ and $k_{cat}^{C}$ in the linear regression. The dashed lines were obtained by optimizing both the coefficient and intercept in the linear regression. Deviation of the dashed line from the solid line is statistically significant (with non-zero intercept, P< 0.01). From Equation 2, the intercepts are the product of the expected (estimation of the mean) values < *k_-m_* > and the corresponding slopes (1/< *K_R_k_6_*>). If the intercept is non-zero, < *k_-m_* > must also be non-zero, indicating the high likelihood of decarboxylation and deoxygenation in a significantly large number of Rubiscos. Furthermore, we have reproduced the trade-off between *S_C/0_* and $k_{cat}^{O}$ **(C)**, from the data of Savir et al. (2010) used to demonstrate “optimality in a low dimensional landscape”; this shows that it is not mirrored when a larger sampling of data for plant Rubiscos is used.

(1) $$\gamma_{C}=k_{3}\left(k_{8}+k_{9}\right)/\left\{k_{7}\left(k_{3}+k_{9}\right)+k_{3}\left(k_{8}+k_{9}\right)\right\},\;$$

where *k*_9_ is the rate of product release, *k*_3_ rate of enolization, and *k*_7_ rate of product formation (*k*_7_/*k*_8_ is the equilibrium constant for enzyme-bound product formation). If only product release (*k*_9_) is rate limiting and the *k*_7_/*k*_8_ equilibrium is achieved rapidly (*k*_9_≪*k*_8_) then *γ_C_*=*k*_8_/(*k*_7_+*k*_8_) must be a relatively small number (<< 1) as equilibrium strongly favors formation of the product. However, there is no evidence that product release limits the reaction, so we need not consider it further. If, as expected, hydration/cleavage (*k*_7_) is rate limiting, or co-limits with enolization (*k*_3_) then clearly *γ_C_*≈1 and consequently, it is erroneous to suggest (Tcherkez et al., 2018) that “ *γ_C_* must be a relatively small number.” Exactly the same arguments apply to the oxygenation reaction, and thus *γ_O_*≈1. Secondly, we do not “disregard conditions of validity to perform a Taylor expansion.” The linear equation is clearly a reasonable approximation (Equation 1, **Figures 2A, B** in Cummins et al. 2018a) for Rubiscos with low enough *k_cat_*, i.e., where higher-order terms in the expansion diminish. We do not make any claim that the expected values of kinetic constants estimated in this way are valid where the variation in *K_C_* becomes rapid as *k_cat_* increases. We address the second question raised in **Table 1** in Tcherkez et al. (2018) as follows:

***Is enolization variable and thus can K_R_ (and γ_C _) change a lot between Rubiscos?*** We agree that the answer to this question in **Table 1** in Tcherkez et al. (2018) is “yes,” but this is precisely why statistical analysis should be applied. Consequently, the two points raised in relation to this question seem to us irrelevant. The “constant” values for the coefficients obtained from the regression analysis do not imply, as Tcherkez et al. (2018) seem to suggest, constant values for the underlying rate constants which we all agree will obviously vary among Rubiscos. It must be emphasized that the coefficients (together with their confidence intervals) should be interpreted as an estimation of a population mean (expected value) of rate constants (or functions thereof, e.g., 1/*K*_R_ *k*_6_) based on a limited sample of Rubiscos. Thus, the estimation of mean decarboxylation and deoxygenation rates varies over a range of values defined by the confidence intervals (**Table 3**, in Cummins et al., 2018a). While the increase in *K_C_* over the entire range of *k_cat_* appears exponential, variations are likely to be more linear within a given taxonomic group. The main limitation of the regression analysis is the availability of a sample that is representative of the population distribution within a taxonomic group. The fact that linear regression *is* representative of the higher plant data is demonstrated below using a more extensive Rubisco sampling.

## Linear Regression Is Representative of the Data

In essence, Cummins et al. (2018a) is not an attempt to “extract implicit rate constants.” Except perhaps for *k*_7_ if rate limiting), it is impossible to determine rate constants for specific Rubiscos from the kinetic data. Rather, it is sufficient to infer whether linear regression is representative of the data by applying hypothesis testing based on analysis of variance (ANOVA) to the coefficient and intercept, which are both functions of the implicit rate constants according to the kinetic mechanism (Equations 1 and 2 in Cummins et al., 2018a). However, recognizing that the sample sizes may not be representative (**Table 1** in Tcherkez et al., 2018; Cummins et al., 2018a) of the population, we have searched the literature for more data. **Figure 1** illustrates the application of the linear regression analysis to a wider-ranging set of carboxylation and oxygenation data (including **Table 1** from Tcherkez et al., 2018; Cummins et al., 2018a) for wild-type Rubiscos in higher plants assembled from various sources (Galmés et al., 2014; Prins et al., 2016; Orr et al., 2016; Ishikawa et al., 2009). The general equation which relates the regression coefficients to the rate constants can be written as (Equations 1 and 2 in Cummins et al., 2018a, where here for clarity we have made the substitutions *γ_C_*=*γ_O_*=1, *k*_6_=*k_-C _, k*_5_=*k_+C _*, *k*_12_ = *k*_-_ *_O_* and *k*_11_ = *k*_+_ *_O_*)

(2) $$K_{m}=\;(k_{cat}^{m}+k_{-m})/\left(K_{R}k_{+m}\right)$$

Thus *k_+m_* and *k_-m_* are, respectively, the binding and dissociation rate constants for CO_2_ or O_2_. The linear increase in the observed *K_m_* as a function of $k_{cat}^{m}$ is clearly apparent in **Figure 1** (P < 0.01 for the coefficient). Note, however, that the carboxylation data (**A**) exhibit a very high degree of heteroscedasticity, with the residuals expanding as $k_{cat}^{C}$ increases, indicating increasing variance in *k_-C_* and *K_R_k_+C_* and specificity, $S_{C}=k_{cat}^{C}/K_{C}$. If there is negligible dissociation ( $k_{-m}\ll k_{cat}^{m}$ for all wild-type Rubiscos), the variation in *K_m_* can only arise from *K_R_k_+m_*. If there is significant CO_2_ and O_2_ dissociation, the scatter in the plots derives from variations in both *k_-m_* and *K_R_k_+m_*. From the results in **Figure 1**, it is more likely than not that the intercept of the regression line (dashed lines) is non-zero (P < 0.01), indicating decarboxylation and deoxygenation do have an effect on *K_m_*. It is important to note, however, due to possible variations between $k_{cat}^{m}$ and *k_-m_* that the influence of decarboxylation and deoxygenation on kinetic behavior does not necessarily apply to all Rubiscos. Consequently, we might expect to see a range of commitments, or partitioning between reaction intermediate and product, *p* (McNevin et al., 2007),

(3) $$p=k_{cat}^{m}/\left(\;k_{cat}^{m}+k_{-m}\right)$$

in wild-type plants ranging from perhaps as low as 50–60% up to 90–100%, depending on the effect of sequence variation on the rates of gas dissociation and catalysis.

## Experimental Evidence Against Decarboxylation and Deoxygenation

Notwithstanding that our analysis does suggest negligible rates of decarboxylation and deoxygenation are likely to be found in many Rubiscos, we feel it appropriate to comment on the experimental evidence put forward by Tcherkez et al. (2018). The “direct evidence” against deoxygenation and decarboxylation (**Table 1** in Tcherkez et al. (2018) is in reality interpretations of various experiments and not definitive experimental findings. We address each of the points raised under the first and third questions in **Table 1** in Tcherkez et al. (2018) as follows:

### Is the Decarboxylation Rate of Importance?

***Point 1*** **:** Hydrolysis of the isolated reaction intermediate 3-keto-2′-carboxyarabinitol-1,5-bisphosphate (CKABP), which is relatively stable in solution, proceeds without significant decarboxylation, although its catalytic rate is poor (slower by a factor of ∼50) compared with RuBP (Lorimer et al., 1986). Lorimer et al. (1986) suggest the slower catalysis for CKABP may be due to a conformational change (presumably leaving decarboxylation unaffected). It should be noted, however, that to mimic the actual reaction, the enzyme–substrate complex should also be in an appropriate protonation state (Cummins et al., 2018b). Subsequent to enolization of RuBP, the proton on the O3 carbon of substrate needs to be transferred to the enzyme in order to form the C3-carbonyl group as in CKABP. Thus, post-carboxylation, there is one less proton in the CKABP intermediate compared with RuBP (carbonyl O3 vs hydroxyl O3), and the enzyme active site has one additional proton, most probably on HIS294 (Tcherkez et al., 2013; Cummins et al., 2018b), compared with the state at RuBP binding. Coincidentally, the tight-binding inhibitor (2CABP) found in most crystal structures of Rubisco also has a C3-hydroxyl group as in RuBP and binds to the Rubisco active site in the right conformation despite having the C2-carboxylate group. The proton exchange between the intermediate compound and enzyme during the natural forward reaction would need to be reversed for the backward reaction from CKABP to RuBP to proceed. However, there is apparently an incompatibility when the starting state of the enzyme (without the additional proton to mimic the transaction during enolization) binds the reaction intermediate CKABP, which is probably responsible for slowing down the forward processing of CKABP by up to a factor of 50 (Lorimer et al., 1986) and may also inhibit decarboxylation. Thus, the experiments of Lorimer et al. (1986) are in essence evidence for negligible decarboxylation in CKABP, not for dissociation of CO_2_ in carboxylated RuBP. Nevertheless, if the assumptions of Lorimer et al. (1986) are correct, their findings for one plant Rubisco (spinach) and the inferences drawn from regression analysis (**Figure 1**) are not necessarily inconsistent, as the latter does not preclude potentially many other plant Rubiscos with negligible decarboxylation.

***Point 2:*** Interpretation of the measured kinetic isotope effects in Rubisco (*α*_Rubisco_) in terms of the partitioning for CKABP, *p* (Equation 3), using the equation (McNevin et al., 2007),

(4) $$\alpha_{\text{Rubisco}}\approx \alpha_{\text{carb}}\left(p+\left(1-p\right)/\alpha_{\text{decarb}}\right),$$

relies heavily on knowledge of the intrinsic isotopic ratios for both the carboxylation (*α*_carb_) and decarboxylation (*α*_decarb_) rate constants, which cannot be measured directly and have been variously assumed or estimated: *α*_decarb_=1.07 (McNevin et al., 2007, Tcherkez et al., 2018) or *α*_decarb_=1.04 (Tcherkez et al., 2013).

### Is the Deoxygenation Rate of Importance?

***Point 1:*** Our recent computational study (Kannappan et al., 2019) suggests that rates of catalysis and deoxygenation may well be very similar. The overall reaction, i.e., to products, is certainly highly exothermic, so it clearly cannot be reversed. However, this should not be confused with the first part of the reaction involving binding of the oxygen to the enediolate of RuBP (a determinant of *K_O_*), which was found to be only moderately exothermic—in contrast to the assertion of Tcherkez—thus permitting the possibility of dissociation rates comparable with catalytic rates. As elaborated next in Point 2, the peroxo adduct formed by the hitherto unknown mechanism reported by Kannappan et al. is a strongly stabilized anion with structure (e.g., bond lengths) very different from isolated peroxides. Thus, references to peroxides in general are not relevant; a thermodynamically stable peroxo adduct would have too high a barrier for progression to the next step.

***Point 2:*** The argument that electron spin should explain negligible deoxygenation is ill-conceived because the reverse, i.e., oxygenation, the binding of oxygen to the enediolate of RuBP, must also be spin forbidden. It is possible that both binding and dissociation are feasible by flipping between singlet and triplet states where the energy surfaces crossover (intersystem crossing). This has been verified by our quantum chemical calculations (Kannappan et al., 2019). Note that this is the first study in the literature that has addressed directly how the formally spin-forbidden oxygenation step (triplet O_2_ to singlet peroxo adduct) can be achieved. The mechanism, via a caged biradical enediolate–O_2_ complex, indicates Rubisco is a unique type of oxygenase without precedent in the literature.

***Point 3:*** As discussed for decarboxylation above, intrinsic isotopic ratios are often uncertain.

In summary, there are no direct and conclusive measurements of decarboxylation and deoxygenation rate constants, only inferences drawn from other experimental results.

## Conclusions

Although precise determinations of both decarboxylation and deoxygenation rate constants for specific Rubiscos are non-existent, our inferential statistical analysis (**Figure 1** and Cummins et al., 2018a) of the available kinetic data for higher plants suggests that a significant number of wild-type Rubiscos (but by no means all) likely exhibit decarboxylation and/or deoxygenation that will have a significant negative impact on commitment of CKABP to form product (Equation 3). The results (**Figure 1**) clearly demonstrate that linear regression is representative of the higher-plant wild types for which there are a relative abundance of published data. As is now apparent in the published literature on Rubisco kinetics, as more data come to light, levels of variance far greater than previously expected (Tcherkez et al., 2006; Savir et al., 2010) are now being found. From our analysis based on a sample of approximately 150 Rubiscos (**Figure 1**), the population of higher plants is expected to exhibit a wide range of decarboxylation behavior. Further research is necessary not only into the diversity in higher-plant Rubisco kinetics but also into the kinetics for a wider variety of Rubisco taxonomic forms, which is currently lacking (Hanson, 2016). For the design of efficient Rubiscos, it is important to gain a comprehensive understanding of the nature of the constraints that cause the apparent trade-off in carboxylation rate and relative specificity (*S_C/O_*). The recent directed-evolution studies (Gomez-Fernandez et al., 2018; Wilson et al., 2018) that have reported deviations from this trade-off provide one avenue in this direction. Moreover, it appears that higher plants are not so constrained, as the trade-off is not clearly evident from a much broader sample (**Figure 1**). This conclusion is supported more broadly by a comprehensive statistical analysis of all the kinetic data (Flamholz et al., 2018, Flamholz et al., 2019). However, there are currently insufficient data for all non-plant Rubiscos to be able to judge whether these findings can be generalized. A more precise explanation of Rubisco catalysis, possibly in terms of sequence variation in the preorganization of charged, polar, and nonpolar groups (Warshel et al., 2006; Frushicheva et al., 2014; Jindal and Warshel, 2017), may be required to rationalize these disparate experimental results. As we have already discussed (Cummins et al., 2018a), such kinetic behavior may well be explained by selection mechanisms (Studer et al., 2014), rather than by the interdependence of implicit rate constants (Tcherkez et al., 2006, Tcherkez et al., 2018). The assertion (Tcherkez et al., 2018) that Lys166 can be used to rationalize the interdependence of rate constants is not supported by our recent study (Cummins et al., 2019) on the final stereospecific protonation step of the carboxylase reaction.

## Author Contributions

PC, BK, and JG prepared the response and approved it for submission.

## Conflict of Interest

The authors declare that the research was conducted in the absence of any commercial or financial relationships that could be construed as a potential conflict of interest.
